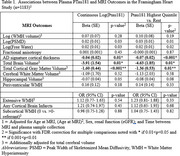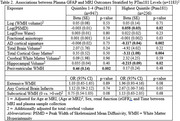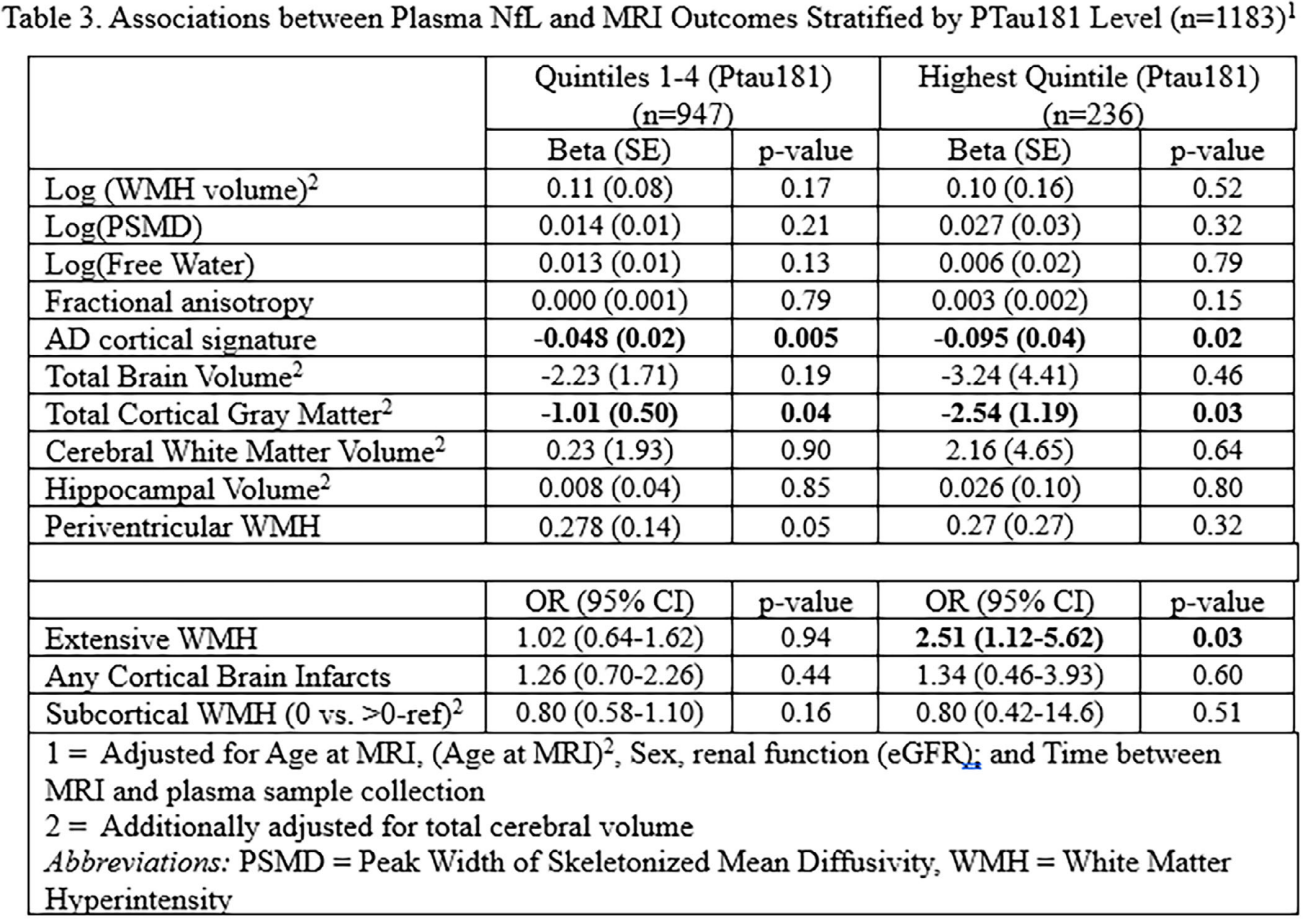# Plasma Ptau as a Biomarker of Structural Brain Health in the Community

**DOI:** 10.1002/alz70862_110179

**Published:** 2025-12-23

**Authors:** Jeremy A. Tanner, Sophia Lu, Hugo J. Aparicio, Sara Doyle, Mitzi M. Gonzales, Jayandra Jung Himali, Tiffany F. Kautz, Terrie‐Jeanne Liu, Pauline Maillard, Emer McGrath, Jaime Ramos‐Cejudo, Claudia L Satizabal, Russell P. Tracy, Mohamad Habes, Suzanne E. Schindler, Charles Decarli, Alexa S Beiser, Sudha Seshadri

**Affiliations:** ^1^ University of Texas Health San Antonio, San Antonio, TX USA; ^2^ Boston University, Boston, MA USA; ^3^ Boston University School of Medicine, Boston, MA USA; ^4^ Cedars‐Sinai Medical Center, Los Angeles, CA USA; ^5^ University of California, Davis, Davis, CA USA; ^6^ University of Galway, Galway, Galway Ireland; ^7^ NYU School of Medicine, New York, NY USA; ^8^ University of Vermont, Colchester, VT USA; ^9^ Washington University in St. Louis, St. Louis, MO USA

## Abstract

**Background:**

Plasma ADRD biomarkers are emerging as accessible and cost‐effective diagnostic tools, but data on their relevance in the general population is needed prior to widespread use. Plasma Ptau shows potential for AD screening, yet its association with structural brain changes in community‐based populations remains unclear. Similarly, it is unclear if plasma NfL and GFAP more closely reflect vascular disease or neurodegeneration, and whether this differs in those with, versus without, AD. This study assesses:(1) the association between plasma Ptau181 and brain MRI outcomes and(2) whether elevated Ptau181 modifies the relationship between NfL, GFAP, and brain MRI features in the Framingham Heart Study(FHS), a flagship population‐based cohort.

**Method:**

FHS Offspring and Omni 1 Cohort participants (Exam 9;2011‐2014) with available plasma biomarkers, brain MRI, and no confounding neurologic disorders were included. Plasma Ptau181, GFAP, and NfL were measured using Quanterix Simoa. MRI outcomes included measures of vascular disease, neurodegeneration, AD‐pattern atrophy, and white matter disease. Plasma Ptau181 was analyzed as a continuous and binary predictor (highest quintile vs remainder) using multivariate linear regressions adjusted for age, age^2^, sex, eGFR, and MRI‐plasma collection interval, with FDR correction. Additional models assessed GFAP and NfL as continuous predictors, stratified by Ptau181 level (highest quintile vs remainder). Sensitivity analyses excluded participants with dementia and/or stroke.

**Result:**

1183 participants were included (mean age 69±8, 56%Female, mean MMSE 29±1.6). Elevated Ptau181 was associated with AD‐pattern cortical thickness in primary and all sensitivity analyses, and with cortical atrophy in primary though not all sensitivity analyses (Table 1). Elevated GFAP was most strongly associated with worsened AD‐pattern atrophy and cortical atrophy in individuals with elevated Ptau181, and with periventricular white matter hyperintensities(WMH) in those with low Ptau181(Table 2). Elevated NfL was associated with AD‐pattern and cortical atrophy in those with/without elevated Ptau181, and additionally with extensive WMH in those with elevated PTau181(Table 3).

**Conclusion:**

The combination of plasma Ptau181, GFAP, and NfL provide information on the etiology and severity of brain disease in the community. In AD, each are associated with worsening neurodegeneration severity, and NfL is also associated with vascular disease. In individuals with low Ptau181, GFAP is associated with vascular disease.